# Community structure analysis of rejection sensitive personality profiles: A common neural response to social evaluative threat?

**DOI:** 10.3758/s13415-018-0589-1

**Published:** 2018-04-12

**Authors:** Elise D. Kortink, Wouter D. Weeda, Michael J. Crowley, Bregtje Gunther Moor, Melle J. W. van der Molen

**Affiliations:** 1 Developmental and Educational Psychology, Institute of Psychology, Leiden University, Leiden, The Netherlands; 2 Methodology and Statistics, Institute of Psychology, Leiden University, Leiden, The Netherlands; 3 Yale Child Study Center, Yale University, New Haven, CT USA; 4 Leiden Institute for Brain and Cognition, Leiden, The Netherlands

**Keywords:** Attachment, Fear of negative evaluation, Network analysis, Social evaluation, Theta oscillations

## Abstract

Monitoring social threat is essential for maintaining healthy social relationships, and recent studies suggest a neural alarm system that governs our response to social rejection. Frontal-midline theta (4–8 Hz) oscillatory power might act as a neural correlate of this system by being sensitive to unexpected social rejection. Here, we examined whether frontal-midline theta is modulated by individual differences in personality constructs sensitive to social disconnection. In addition, we examined the sensitivity of feedback-related brain potentials (i.e., the feedback-related negativity and P3) to social feedback. Sixty-five undergraduate female participants (mean age = 19.69 years) participated in the Social Judgment Paradigm, a fictitious peer-evaluation task in which participants provided expectancies about being liked/disliked by peer strangers. Thereafter, they received feedback signaling social acceptance/rejection. A community structure analysis was employed to delineate personality profiles in our data. Results provided evidence of two subgroups: one group scored high on attachment-related anxiety and fear of negative evaluation, whereas the other group scored high on attachment-related avoidance and low on fear of negative evaluation. In both groups, unexpected rejection feedback yielded a significant increase in theta power. The feedback-related negativity was sensitive to unexpected feedback, regardless of valence, and was largest for unexpected rejection feedback. The feedback-related P3 was significantly enhanced in response to expected social acceptance feedback. Together, these findings confirm the sensitivity of frontal midline theta oscillations to the processing of social threat, and suggest that this alleged neural alarm system behaves similarly in individuals that differ in personality constructs relevant to social evaluation.

**Electronic supplementary material:**

The online version of this article (10.3758/s13415-018-0589-1) contains supplementary material, which is available to authorized users.

The emotional experience of social rejection can be extensive and painful, (Eisenberger & Lieberman, 2004) and chronic rejection by others has been linked with mental health problems like depression, anxiety, and substance abuse (Rubin, Bukowski, & Parker, 2006). Therefore, the ability to quickly detect and evaluate cues that convey social disconnection in our environment plays an important role in safeguarding psychological health (Baumeister & Leary, 1995). Neuroimaging studies indicated that our brain is equipped with a neural “alarm system” that quickly detects cues communicating social threat (Eisenberger & Lieberman, 2004). Recently, it has been shown that unexpected rejection feedback from peers—a salient threat to social connectedness—elicits a significant increase in frontal-midline theta (FM-theta) oscillatory power (4–8 Hz) (van der Molen, Dekkers, Westenberg, van der Veen, & van der Molen, 2017). This work has been particularly valuable for delineating the neural correlates of the alarm system implicated in social threat. However, it is unclear how FM-theta power responds in individuals who are susceptible to social rejection. Such knowledge is of critical importance to understand the pathogenesis of psychopathology for which social rejection is likely to have transdiagnostic implications. Therefore, the goal of the current study is to examine whether event-related theta power reactivity to social evaluative feedback might depend on individual differences in personality constructs relevant to social disconnection.

Individual differences in the response to social threat have been observed in previous studies (DeWall et al., 2012; Mikulincer & Shaver, 2007; Somerville, Kelley, & Heatherton, 2010). Specifically, attachment-related anxiety and avoidance are thought to influence the way we respond to social evaluation and social threat (DeWall et al., 2012; Mikulincer & Shaver, 2007). Rooted in early childhood, our attachment style drives our behavior from our innate need to form close, lasting bond with others (Baumeister & Leary, 1995). People high in attachment-related anxiety show more intense behavioral and affective responses to social threat (Gillath, Bunge, Shaver, Wendelken, & Mikulincer, 2005). In contrast, people high in attachment-related avoidance are thought to disengage themselves from interpersonal relations, leading to a blunted reaction to social rejection (Campbell, Simpson, Boldry, & Kashy, 2005; Fraley & Brumbaugh, 2007). Indeed, research shows that avoidantly attached people display reduced reactivity in the anterior cingulate cortex and anterior insula following social exclusion (DeWall et al., 2012; Mikulincer, 2016; Vrticka, Bondolfi, Sander, & Vuilleumier, 2012). These studies seem to suggest a dissociation in reactivity of the alleged neural alarm system in individuals high in attachment-related anxiety relative to individuals high in attachment-related avoidance (Eisenberger & Lieberman, 2004). Although these important studies underscore the relevance of individual differences in biological responses to social disconnection, it remains unclear whether individual differences in personality constructs related to social connectedness modulate the reactivity of the neural alarm system as indexed by theta power dynamics.

Cognitive neuroscience studies have provided strong evidence that FM-theta oscillations play an important role in orchestrating cognitive control operations when processing cues that convey errors and punishment (Cavanagh & Frank, 2014). It has been suggested that FM-theta reflects the “need for control” after cognitive conflict, which typically occurs whenever there is uncertainty about an optimal course of action (Cavanagh, Figueroa, Cohen, & Frank, 2012; Cohen & Donner, 2013). FM-theta seems to be generated by a broad cingulate network of which the dorsal anterior cingulate cortex has been posited as the main source (Young & McNaughton, 2009). The cingulate cortex acts as an important neural hub implicated in cost-benefit analyses determining whenever control efforts are required (Shenhav, Botvinick, & Cohen, 2013; Shenhav, Cohen, & Botvinick, 2016). Recent evidence suggests that such control operations are not restricted to cognitive processes, but extend to nociception and negative affect as well (Shackman et al., 2011; Vogt, 2016). For example, according to the adaptive control hypothesis, (Shackman et al., 2011) the mid-cingulate cortex acts as a common mechanism sensitive to the elicitation of cognitive control, as well as elicitation of pain and negative affect. FM-theta seems to reflect an electrophysiological mechanism of these domain general processes (Cavanagh & Shackman, 2015; Shackman et al., 2011). Indeed, recent studies have pointed towards the relevance of FM-theta oscillations in the processing of cues communicating negative affect, such as social rejection (van der Molen et al., 2017). This FM-theta power reactivity in response to social threat seems to be governed by two important structures implicated in saliency detection and conflict monitoring—the anterior cingulate cortex and anterior insula (Cristofori et al., 2013; van der Molen et al., 2017).

Here we will examine whether individual differences in personality constructs related to social evaluation (e.g., attachment related anxiety vs. avoidance, fear of negative/positive evaluation, self-esteem) are reflected in FM-theta power reactivity to unexpected social rejection. Although these personality constructs have been studied separately in studies on social evaluation (DeWall et al., 2012; Somerville et al., 2010; van der Molen et al., 2017; Vrticka et al., 2012), to our knowledge, no studies have jointly examined these constructs in relation to the neural correlates of social evaluative feedback processing. In the current study, we employed the Social Judgment Paradigm (SJP; van der Molen et al., 2017; van der Molen et al., 2013) to examine the neural reactivity to social rejection and acceptance feedback. In this paradigm, participants were led to believe they had been evaluated by a group of peers, who indicated whether they would like/dislike the participant. Participants predicted during the experiment whether each peer had liked/disliked them. After each prediction, participants received peer feedback indicating social acceptance or rejection. Since peer feedback could either be congruent or incongruent with participants’ prior predictions, this task allowed for discriminating between the effects of feedback valence and congruency on theta-power reactivity.

To examine individual differences in FM-theta power reactivity to social evaluative feedback processing, we used a community structure detection analysis to dissect profiles (or subgroups) based on participants’ scores on the personality constructs relevant to social evaluative distress (e.g., anxious vs. avoidant attachment, fear of negative/positive evaluation, and self-esteem). We hypothesized that the distinction between an anxious versus avoidant tendency toward social evaluation would be expressed by the differential clustering of scores on these personality constructs. This notion of clustering of psychological traits (or symptoms) within and between individuals has gained popularity in the scientific community (Fair, Bathula, Nikolas, & Nigg, 2012; Sonuga-Barke, Bitsakou, & Thompson, 2010). Most critically, these methods help in clarifying individual differences in the etiology and treatment of psychopathology (Agid et al., 2007). Thus, network theory might be particularly suited to examine the expected anxious versus avoidant profiles in our data set. It was anticipated that community structure analysis would yield two distinct subgroups that could be interpreted as an anxious subgroup (i.e., scoring high on attachment-related anxiety, fear of negative/positive evaluation, and low on self-esteem and attachment-related avoidance), and an avoidant subgroup (i.e. scoring high on attachment-related avoidance and self-esteem, and low on fear of negative/positive evaluation and attachment-related anxiety) (Clark & McManus, 2002; DeWall et al., 2012; Fraley & Brumbaugh, 2007; Guyer et al., 2008; Vrticka & Vuilleumier, 2012; Weeks, Heimberg, & Rodebaugh, 2008). We predicted that unexpected rejection feedback would induce a significant increase in theta power (van der Molen et al., 2017). Based on prior neuroimaging results indicating hypersensitive versus hyposensitive responsivity of the anterior cingulate cortex and anterior insula to social threat (Eisenberger, 2012; Rotge et al., 2015), we expected that FM-theta power to unexpected rejection feedback would be significantly higher in the anxious subgroup than in the avoidant subgroup. Although our a priori hypotheses were directed towards FM-theta sensitivity in this study, based on prior results (cf. van der Molen, Dekkers, Westenberg, van der Veen, & van der Molen, 2017), we additionally examined the feedback-related brain potentials commonly observed using this paradigm: the feedback-related negativity (FRN) and P3. We expected that the FRN would be sensitive to unexpected feedback, whereas the P3 would be sensitive to expected acceptance feedback (van der Molen et al., 2017; van der Molen et al., 2013; van der Veen, van der Molen, Sahibdin, & Franken, 2014).

## Method

### Participants

Seventy-eight undergraduate students took part in this study. Only female students were included, since previous research has uncovered that females show greater responses to social rejection than males (Stroud, Salovey, & Epel, 2002). Thirteen participants were excluded from further analysis due to disbelief in the cover story of the SJP (*n* = 9), recording problems (*n* = 1), or bad EEG data (*n* = 3), leading to a total sample of 65 participants for data analyses (age range = 18–24 years, *M* = 19.69, *SD* = 1.45). Participants were recruited from or within proximity of Leiden University. They provided signed informed consent prior to the experiment and received course credit or fixed payment for participation. Participants were right-handed, had normal or corrected-to-normal vision, and did not use psychoactive medication. Further exclusion criteria entailed extensive drug or alcohol use, a neurological disorder, and brain trauma. The protocol of this study was approved by the local Ethics Committee of the Institute of Psychology of Leiden University.

### Self-report personality questionnaires

#### Attachment

The Experiences in Close Relationships scale (ECR; Brennan & Morris, 1997) was administered to determine the participants’ attachment-related anxiety and attachment-related avoidance score. The ECR includes 36 items, of which 18 index the anxiety dimension and 18 index the avoidance dimension. Participants filled out the ECR twice—both during recruitment approximately 1 month before the experimental task, and on the day of the experiment. The ECR requires participants to indicate the extent to which statements about cognitive, emotional, and behavioral patterns in romantic partner relationships apply to them, on a 7-point Likert scale, ranging from 1 (*not at all*) to 7 (*very much*) (Brennan, Clark, & Shaver, 1998). Previous research has proven the reliability and validity of the ECR to be satisfactory and its internal consistency to be good; Cronbach’s alpha was .88 for the attachment-related anxiety dimension and .90 for the attachment-related avoidance dimension (Frias & Shaver, 2014).

#### Fear of negative evaluation

The Brief Fear of Negative Evaluation Scale–Revised (BFNES-R; Carleton, McCreary, Norton, & Asmundson, 2006) (BFNES-R) was used to measure fear of negative evaluation. The BFNES-R consists of 12 items, with a 5-point Likert scale, ranging from 0 (*not at all characteristic of me*) to 4 (*extremely characteristic of me*). Higher total scores on the BFNES-R are reflective of high concern with other people’s social evaluation, high approval seeking, avoiding other people’s disapproval, and avoiding social-evaluative situations (Watson & Friend, 1969). Internal consistency was found to be excellent, with Cronbach's alpha’s between .89 (Carleton et al., 2006) and .97 (Carleton, Collimore, McCabe, & Antony, 2011).

#### Fear of positive evaluation

The Fear of Positive Evaluation Scale (FPES; Weeks et al., 2008) was used to provide an index of whether participants’ fear being positively and publicly evaluated. This questionnaire was used to validate that the anticipated heightened response to unexpected social rejection feedback was related to individual differences in fear of *negative* evaluation, and not social evaluation in a broader sense. The FPES consists of 10 items and uses a 10-point Likert scale, ranging from 0 (*not at all true*) to 9 (*very true*). Higher total scores relate to a higher fear of positive evaluation. The instrument has previously been proven to have sufficient internal consistency, with Cronbach’s alpha’s of .80 or higher, as well as good test–retest reliability (ICC = .70). The construct of FPE is distinct from, but strongly correlated with, fear of negative evaluation (Weeks et al., 2008).

#### Self-esteem

The 10-item Rosenberg Self-Esteem Questionnaire (RSEQ; Rosenberg, 1965) was used to measure participants’ levels of self-esteem and self-worth. Participants are required to answer on a 4-point Likert scale, ranging from 1 (*strongly agree*) to 4 (*strongly disagree*). Lower self-esteem levels are reflected by a lower total score, and a cut-off score of 15 is thought to be an indication of low self-esteem. The RSEQ was found to have sufficient reliability and validity, and acceptable to good internal consistency, with Cronbach’s alpha’s ranging from .77 to .88 (Rosenberg, 1965).

### Social Judgment Paradigm

The Social Judgment Paradigm (SJP) was used to examine the electrocortical reactivity to social evaluative peer feedback (cf., van der Molen et al., 2017). Via a cover story, participants were told that they would participate in a study on first impressions. At least a week before the experiment, participants were required to send a personal portrait photograph to the experimenters. Allegedly, this photograph would be evaluated by a panel of peers from another university. These peers would judge whether they liked or disliked the participant, based on their first impression of the photograph. At least one week later, participants were invited to the lab and participated in the SJP. Participants were informed that they would view portrait photographs of each member of the peer panel that had evaluated their photograph. Prior to the task, participants were asked to indicate to what extent they expected to be liked by the peers in the panel. They could indicate their expectation by placing a mark on a 10-cm line, ranging from 0% to 100% (higher percentages indicated more positive feedback expectancies). After the task, participants were asked to indicate in a similar fashion the percentage of social acceptance feedback they received. During the task, participants were required to indicate whether they expected the peer on each photograph had liked or disliked them. After a fixed anticipation period, participants were presented with actual peer feedback communicating social acceptance/rejection that was congruent or incongruent with participants’ prior predictions (like/dislike). This resulted in four possible task conditions: expected acceptance, expected rejection, unexpected acceptance, and unexpected rejection. In reality, participants had not been evaluated by a panel of peers, but feedback was computer generated.

**Fig. 1:**
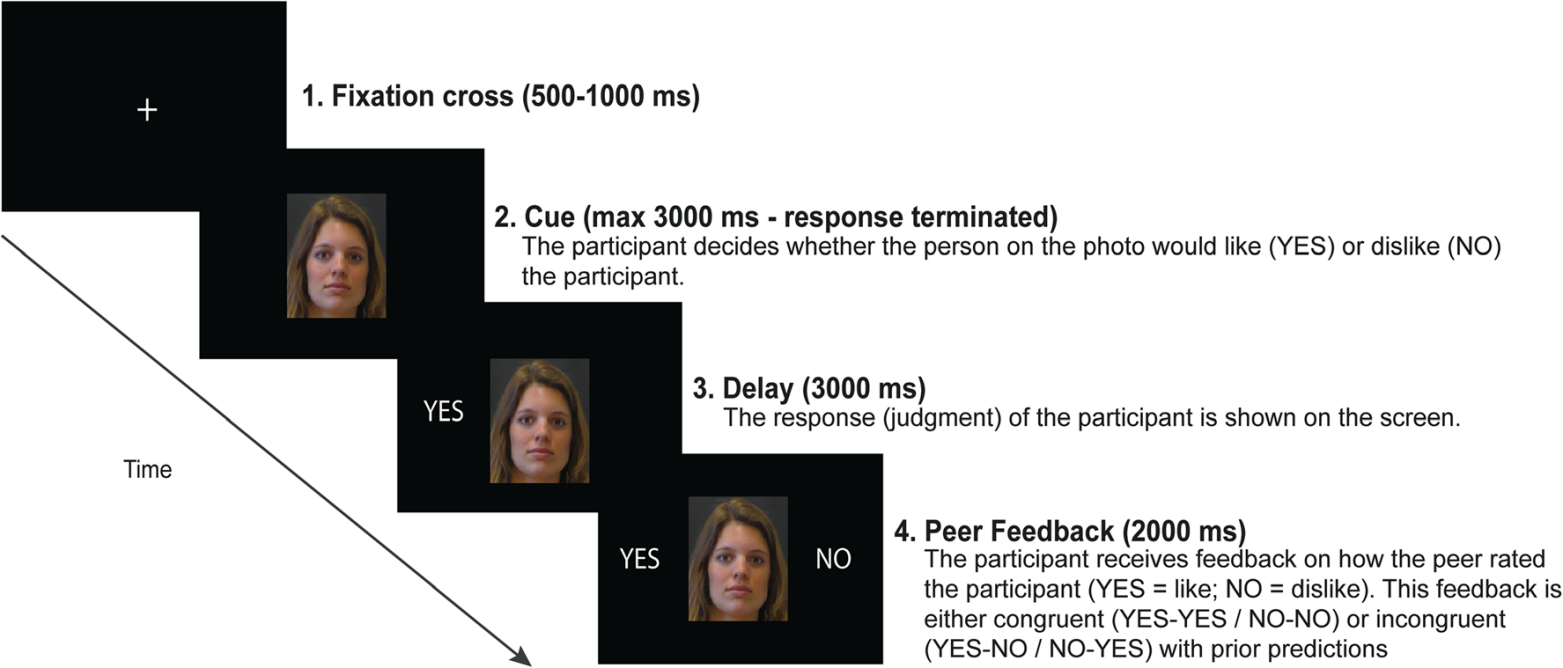
Schematic overview of a single trial sequence of the social judgment paradigm. Reprinted from NeuroImage, 146, Van der Molen, M.J.W.,Dekkers, L.M.S., Westenberg, P.M., Van der Veen, F.M., &Van der Molen, M.W., Why don't you like me? Midfrontaltheta power in response to unexpected peer rejectionfeedback, 474–783, Copyright (2017), with permission from Elsevier

The SJP consisted of 160 trials that started with a fixation cross (jittered duration of 500–1,000 ms) followed by the presentation of the peer photograph that was presented on the screen during the remainder of the trial. Peer photos had a neutral facial expression (as determined with the Self-Assessment Manikin; Bradley & Lang, 1994) and were obtained during previous studies (Gunther Moor, Van Leijenhorst, Rombouts, Crone, & Van der Molen, 2010; van der Molen et al., 2013). Presentation of the peer photos consisted of the following parts: cue, delay, and feedback. During the cue part, participants had to indicate whether they thought this peer had liked or disliked them. Participants could communicate their prediction by pressing one of two buttons on the armrests (left vs. right) of their chair. One button corresponded to expected social acceptance (“YES,” this peer liked me), the other to expected social rejection (“NO,” this peer did not like me). This button-type versus prediction-type connotation was counterbalanced between participants. Participants had 3,000 ms to provide their prediction starting with the onset of the cue. If they did not manage to respond within this time window, the words “too slow” appeared on the monitor (5,000-ms duration), followed by a new trial. If they did respond on time, participants’ predictions (“YES” or “NO”) were presented on the computer screen (left side of the cue) for 3,000 ms. Thereafter, peer feedback (“YES” = like; “NO” = dislike) was presented to the right side of the cue (2,000-ms duration). Feedback was generated by the computer in pseudorandom order. Participants received social acceptance feedback on 50% of the trials. The first 10 trials on the SJP were practice trials. The remaining 150 trials were presented in three blocks of 50 trials each. The photos were presented with E-Prime 2.0 software (Psychology Software Tools, Pittsburgh. PA) and were presented on a 17-inch monitor (60 Hz refresh rate; visual angle [width × height] = 4.66° × 6.05°). Figure 1 depicts a schematic overview of one trial sequence.

### Procedure

At the recruitment stage, participants filled out an online self-report questionnaire to screen for exclusion criteria. To determine the stability of their attachment-related anxiety and attachment-related avoidance score over time, participants filled out the ECR both during the recruitment stage and during their lab visit. A positive Pearson product-moment correlation was found between the two administration time points of both the Avoidance scale, *r*(63) = .88, *p* < .001, and the Anxiety scale, *r*(63) = .75, *p* < .001, showing acceptable to good test–retest reliability.^1^ Participants visited the lab for the experimental procedure. Prior to the EEG experiment, participants filled out the above-mentioned personality questionnaires. Then, participants were seated in front of the computer monitor and EEG equipment was applied. The EEG experiment started with a 5-minute resting-state EEG measurement, during which participants were instructed to sit as still as possible with their eyes closed. Thereafter, participants completed the SJP, followed by a second 5-minute resting-state measurement. EEG equipment was then removed. Participants were debriefed about the experiment, and their belief in the cover story was confirmed.

### Community structure analysis

We expected to find specific data-driven phenotypic subtypes, stemming from groups of people sharing similar outcomes on certain personality constructs relevant to social evaluation (i.e., attachment style, fear of negative evaluation, fear of positive evaluation, and self-esteem). To this end, we used community structure detection (Newman, 2006). This analysis is derived from social network theory, where individuals are seen as “nodes” in a network. Nodes are connected (via so-called edges) if individuals know each other. Community structure detection determines whether there are “communities” containing multiple individuals that are connected to all other members of the community but not with members outside their community. In our case, participants constitute the nodes in a network, but whether they are connected depends on the similarity of their scores on the personality constructs. Community detection would thus try to find communities of participants whose profiles of personality constructs are similar to participants in the same community, but dissimilar to profiles of participants in other communities. The community structure within networks can be detected by means of an algorithm—based on eigenvalues of the modularity matrix—that searches for the optimal value of modularity over possible network distributions (Newman, 2006). Allocation to subgroups was established by subgroup assignment over 200 runs of the modularity matrix algorithm. We established the robustness of the community structure based on the quality index Q, which lies between the range of −0.5 and 1. If Q is positive, the division of the networks in groups exceeds chance level (Newman, 2004). The community structure analysis was performed using R software Version 3.3.2 (R Core Team, 2013) and included participants’ scores on the ECR, BFNES-R, FPES, and RSEQ. Subgroups were interpreted based on the scores of these questionnaires. Community structure analysis was preferred over cluster analysis, since community detection does not require a priori specification of the number of clusters, can be more easily visualized and offers a clear interpretable measure of model fit (i.e., modularity).

### EEG recording and processing

EEG was recorded with a Biosemi Active Two system (Biosemi, Amsterdam, The Netherlands) at a 1024 Hz sampling rate from 64 active scalp electrodes, four ocular electrodes, and two electrodes placed at the left and right mastoids, which served as off-line reference. Vertical eye movements were recorded with two electrodes placed above and below the left eye. Horizontal eye movements were recorded with two electrodes placed at the left and right canthus. The Biosemi system ensures that the ground electrode is replaced by an electric feedback circuit through the common mode sense (CMS) electrode and driven right leg (DRL) electrode, which were used as online reference.

Offline data analysis was performed in Brain Vision Analyzer (BVA 2.0.4; Brain Products GmbH, Munich, Germany). Data were down-sampled to 512 Hz and rereferenced to the average of the left and right mastoids. A 0.5–70 Hz band-pass filter (24 dB/oct) and a 50 Hz notch filter was applied, epochs from −1.5 s to +10 s enclosing the onset of the cue (i.e., photo) were created, followed by a 500–200 ms precue baseline correction. The epochs were semiautomatically inspected for artifacts. Presence of artifacts apart from eye blinks (e.g., muscular activity, clipping, blocking), or invalid responses (e.g., more than one response within the response window, responses out of the response window) resulted in elimination of an epoch from further analysis. Spherical spline interpolation was used to correct bad channels, and eye blinks were removed using the ocular independent component analysis method. Next, epochs of −4 s to +4 s enclosing the feedback stimuli were created, and a current source density (CSD) transformation was applied to the time series. The average number of artifact-free EEG trials that were used for analyses are 37.51 (*SD* = 7.24) for the expected acceptance condition, 34.43 (*SD* = 7.48) for the unexpected rejection condition, 28.95 (*SD* = 7.26) for the expected rejection condition, and 32.12 (*SD* = 8.28) for the unexpected acceptance condition. Trials on which participants did not respond, or responded too late, were omitted from this overview and further analyses. The number of artifact-free trials in the analysis did not vary systematically with any of the measures of interest (*p*s > .05).

### Time-frequency power analyses

Time-frequency characteristics were extracted from the EEG time series by convolution of the single trials with a family of complex Morlet wavelets, which can be defined as Gaussian-windowed sine waves:

$$ \Psi \left(t,f\right)={Ae}^{-{t}^2/\left(2{\sigma}_t^2\right)}\times {e}^{i2\pi ft} $$

were Ψ denotes the complex conjugation with the wavelet function, *t* is time, and *f* is frequency, which increased from 0.5 to 70 Hz in 60 logarithmically spaced steps. *A* represents the wavelet normalization function so that all frequencies have the same energy value of 1, which allows comparing the signal across all frequency levels, and *σ*_t_ represents the standard deviation of the Gaussian bell function. The family of complex Morlet wavelets are defined by the Morlet parameter *C* = *f*(2*πσ*_*t*_), which was set to 5 to obtain an adequate trade-off between time and frequency precision. After convolution of the complex Morlet wavelet with the single trial data, time-frequency power was extracted from the complex signal: *p*(*t*) = (real [*z*(*t*)]^2^ + imag [*z*(*t*)]^2^), and was normalized using a percent-change from baseline (i.e., the −500 to −200 ms prestimulus interval). Theta power from the Fz electrode was analyzed, since peak theta power collapsed over conditions and groups was highest for this lead, and since this facilitates comparisons with prior results (van der Molen et al., 2017).

### Feedback-related brain potentials

The artifact-free segments obtained via the abovementioned procedure were further segmented into 1,200-ms epochs including a 200 ms prefeedback interval that was used for baseline-correction. Extraction of FRN and P3 amplitudes was performed following the procedure as described in our previous studies (Dekkers, van der Molen, Gunther Moor, van der Veen, & van der Molen, 2015; van der Molen et al., 2013). The FRN was calculated via a peak-to-peak detection method in which the amplitude of the P2 component (270–290 ms) was subtracted from the most negative peak that followed the P2 (300–320 ms). This method reduces overlap of other brain potentials surrounding the FRN (Holroyd, Nieuwenhuis, Yeung, & Cohen, 2003). The P3 was determined by calculating the mean amplitude in the 360–460 ms post feedback interval. These amplitude extraction time windows were determined by inspecting the grand-averaged ERP, collapsed over conditions and all subjects (Kappenman & Luck, 2016). Since previous studies using this paradigm have shown that effects of social-evaluative feedback are most prominently observed at the anterior midline for both the FRN and P3(Dekkers et al., 2015; van der Veen et al., 2014), we examined the ERPs at Fz.^2^

### Statistical analysis

Statistical analyses were performed using IBM Statistics (Version 24, IBM Corporation, 1989–2011). First, a bias score was calculated based on the participants’ number of acceptance and rejection predictions, as well as their corresponding reaction time (RT). The bias score was calculated by dividing the amount of acceptance expectancies by the amount of total judgements and suggests either an optimism bias (>50%) or a pessimism bias (<50%)(Dekkers et al., 2015; van der Veen et al., 2014). A one-sample *t* test was performed to check whether this bias score differed significantly from baseline (50%). Next, subgroups derived from the community structure analyses were examined for differences in self-reported personality constructs (ECR, BFNES-R, FPES, RSEQ), via separate one-way ANOVAs. *Z*-score transformations were applied to the questionnaires’ total scores to enable comparison. Also, using one-way ANOVAs, the subgroups were compared on behavioral data of the SJP. Lastly, a mixed-design repeated-measures analysis was performed on log-transformed theta power, with feedback expectancy (two levels: expected, unexpected) and valence (two levels: acceptance, rejection) as within-subject factors, and the number of subgroups as between-subjects factor. Two similar sets of mixed-design repeated-measures ANOVAs were performed for the FRN and P3 results. Bonferroni corrections were applied for multiple testing. Greenhouse–Geisser correction was applied if applicable, but uncorrected degrees of freedom are reported for clarity. Lastly, Pearson product-moment correlation was performed to examine the correlations between theta power responses and self-report measures (ECR, FNE, FPES, and BFNES-R).

## Results

### Community structure analysis

**Fig. 2:**
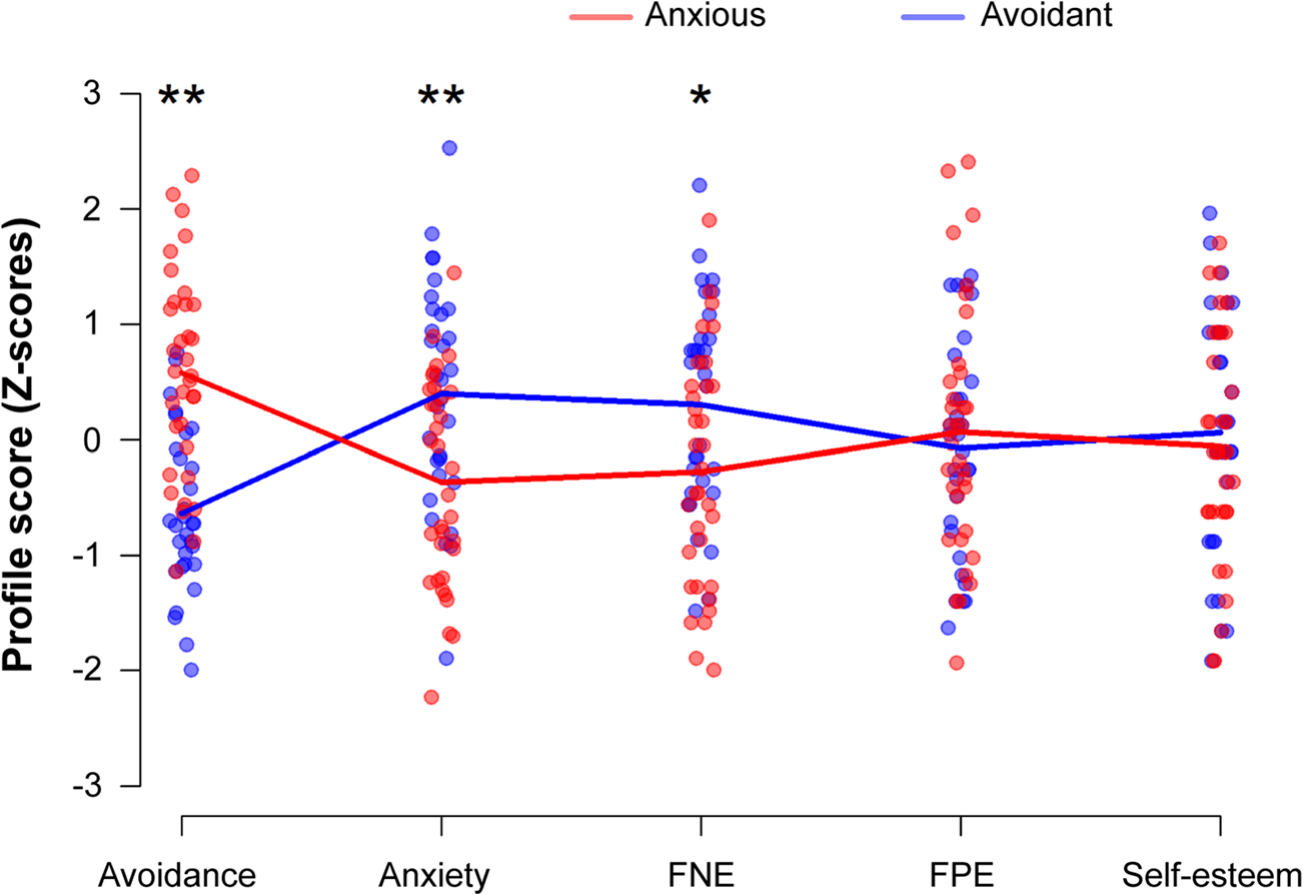
Community-derived subgroups based on the personality constructs (presented on the *x*-axis). Participants’ profile scores (*z* scores) are presented on the *y*-axis. The anxious subgroup (*n* = 31) is indicated in blue and the avoidant subgroup (*n* = 34) in red. FNE = fear of negative evaluation; FPE = fear of positive evaluation.*significant (*p* < .05) mean difference between subgroups. **significant (*p* < .01) mean difference between subgroups. (Color figure online)

The community structure analysis yielded two subgroups within our data set. The modularity quality index (Q = .018) showed that these subgroups differed above chance level, although some overlap between these two subgroups is still present since Q values above .40 are argued to indicate completely distinct subgroups (Fortunato & Barthelemy, 2007). Figure 2 depicts the profile scores of the two subgroups on the personality questionnaires, whereas Table 1 presents the average total scores on these questionnaires.

**Table 1:** Means and standard deviations (*SD*) of the self-reported questionnaires for the anxious subgroup (*n* = 31) and avoidant subgroup (*n* = 34)

	Anxious	Avoidant	Total sample
	Mean (*SD*)	Mean (*SD*)	Mean (*SD*)
ECR: Avoidance**	2.29 (2.17)	3.42 (0.84)	2.88 (0.93)
ECR: Anxiety**	4.02 (0.83)	3.30 (0.77)	3.64 (0.87)
Fear of negative evaluation*	36.45 (8.96)	30.76 (9.78)	33.48 (9.76)
Fear of positive evaluation	32.42 (11.97)	34.32 (14.23)	33.42 (13.14)
Self-esteem	24.65 (4.01)	24.21 (3.76)	24.42 (3.86)

*Note.* ECR = experiences in close relationships. *significant (*p* < .05) mean difference between subgroups. **significant (*p* < .01) mean difference between subgroups

Post hoc testing indicated that these subgroups differed significantly regarding their scores on the ECR subscales, as well as their self-reported level of fear of negative evaluation. One subgroup (*n* = 31), hereafter labeled the anxious subgroup, scored high on both attachment-related anxiety and fear of negative evaluation, and low on attachment-related avoidance. The other subgroup (*n* = 34), hereafter labeled the avoidant subgroup, showed an opposing pattern, with high scores on attachment-related avoidance, but low scores on attachment-related anxiety and fear of negative evaluation. Interestingly, the two subgroups showed minimal differences on fear of positive evaluation and self-esteem. It thus seems that the constructs of attachment-related avoidance and anxiety and fear of negative evaluation explain the subgroups in our sample. Average scores of self-report measures for the total sample are presented in Table 1 (see the Supplementary Data for the behavioral results on the SJP of the total sample).

### Subgroup characteristics on the SJP

Participants in the anxious subgroup showed a bias in their expectancies of the outcome of social evaluation. They predicted social acceptance feedback on 56.03% (*SD* = 8.22) of the trials, which differed significantly from 50%, *t*(30) = 4.09, *p* < .001. Participants in the avoidant subgroup predicted social acceptance feedback on 52% (*SD* = 9.04), which did not differ significantly from 50%, *t*(33) = 1.59, *p* = .12. In the anxious subgroup, response latencies to predict the feedback outcome were significantly longer when they predicted social rejection relative to acceptance (mean difference = 0.36 ms), *t*(31) = 2.05, *p* = .049. No differences in response latencies were observed in the avoidant subgroup (mean difference = 0.28 ms), *t*(34) = 0.98, *p* = .33. These behavioral data are presented in Table 2.

**Table 2:** The average number of trials and response time (ms), and standard deviations (*SD*), during the SJP for the anxious subgroup (*n* = 31) and avoidant subgroup (*n* = 34). Averages are presented for predicted social acceptance feedback (“Yes”) and predicted social rejection feedback (“No”)

	Anxious	Avoidant
Yes		
Number of trials (*SD*)	83.45 (12.08)	78.21 (13.60)
Response time (*SD*)	1411.16 (292.21)	1404.53 (280.05)
No		
Number of trials (*SD*)	65.55 (12.47)	70.82 (13.43)
Response time (*SD*)	1447.80 (298.34)	1432.90 (319.51)

We also asked participants prior to the SJP whether they expected to receive more acceptance or rejection feedback. Both subgroups expected to receive a larger proportion of social acceptance feedback (estimates differed significantly from 50%, *p*s < .01). After the SJP, we asked participants about their recollection of the distribution of acceptance vs. rejection feedback. Both subgroups estimated to have received a slightly larger proportion of rejection feedback (estimates differed significantly from the actual proportion of rejection feedback received, 50%, *p*s < .05). No group differences in these pre/post feedback ratings were found (see Table 3).

**Table 3:** Means and standard deviations (*SD*) of the social acceptance feedback prediction before and after the SJP. Results are presented per subgroup: anxious subgroup (*n* = 31) and avoidant subgroup (*n* = 34)

	Anxious	Avoidant
	Mean (*SD*)	Mean (*SD*)
Acceptance prediction before SJP	67.13 (8.37)**	65.03 (9.85)**
Acceptance prediction after SJP	45.81 (8.94)*	45.45 (10.48)*

*Note.* *significant (*p* < .05) mean difference from 50%. **significant (*p* < .01) mean difference from 50%

### Theta power

**Fig. 3:**
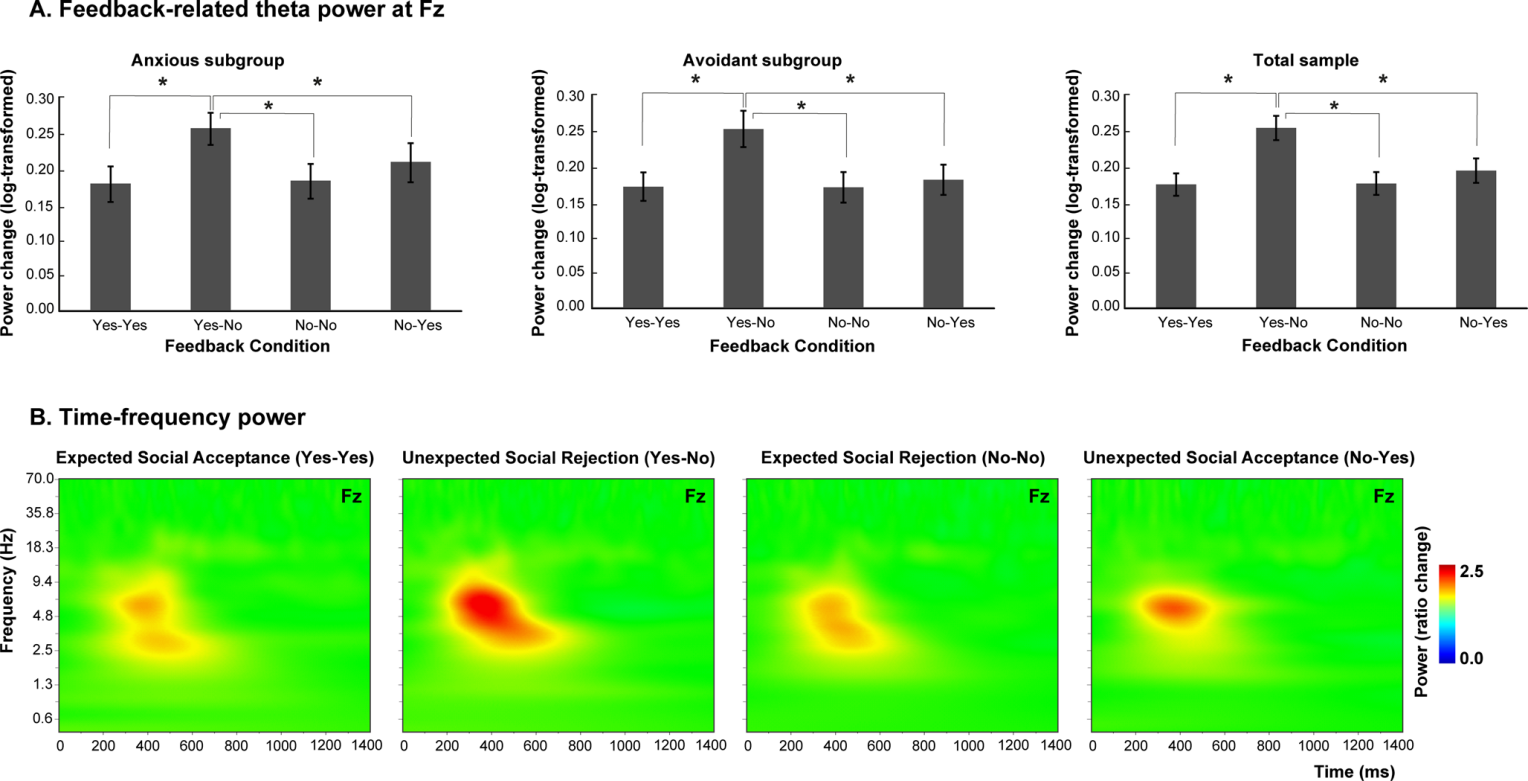
Theta power (4–8 Hz) at Fz. **a** Theta power was significantly higher in the unexpected rejection condition relative to other feedback conditions. This effect was similar for the total sample (*N* = 65) and for the anxious subgroup (*n* = 31) and avoidant subgroup (*n* = 34). Yes-Yes = expected acceptance; Yes-No = unexpected rejection; No-No = expected rejection; No-Yes = unexpected acceptance. Error bars indicate *SEM*. **b** This significant theta power increase during the unexpected rejection condition is displayed in the time-frequency plot. (Color figure online)

The mixed-design ANOVA on theta power generated a main effect of Expectancy, *F*(1, 64) = 4.46, *p* = .039, η_p_^2^ = .066, as well as a main effect of Valence, *F*(1,64) = 6.62, *p* = .027, η_p_^2^ = .012, which were included in a significant Expectancy × Valence interaction, *F*(1, 64) = 21.20, *p* < .001, η_p_^2^ = .25. Post hoc comparisons revealed that theta power was significantly higher in the unexpected social rejection condition (Yes-No) relative to all other conditions (Yes-Yes, *p* < .001; No-No, *p* < .001; No-Yes, *p* = .020), whereas no significant differences between the other feedback conditions were found (*p*s > .05). Additionally, no significant main effect of Subgroup was found, *F*(1, 64) = 0.29, *p* = .59, η_p_^2^ = .005, nor were interaction effects observed for Subgroup × Expectancy, *F*(1, 64) = 0.27, *p* = .60, η_p_^2^ = .004; Subgroup × Valence, *F*(1, 64) = 0.16, *p* = .69, η_p_^2^ = .002; and Subgroup × Expectancy × Valence, *F*(1, 64) = 0.09, *p* = .77, η_p_^2^ = .001. Theta power results are shown in Fig. 3. Lastly, no significant correlations were found between theta power responses and self-report measures (ECR, FNE, FPES, and BFNES-R). These correlations are shown in Table 4.^3^

**Table 4:** Pearson product-moment correlation matrix of the self-reported questionnaires and theta power results

Variables	1.	2.	3.	4.	5.	6.	7.	8.	9.
1. Theta Yes-Yes	–								
2. Theta Yes-No	.559**	–							
3. Theta No-No	.486**	.575**	–						
4. Theta No-Yes	.337**	.336**	.526**	–					
5. ECR: Avoidant	.125	.115	.014	.115	–				
6. ECR: Anxiety	.019	.021	.006	.014	.206	–			
7. FNE	−.008	.205	.054	.174	−.035	.245*	–		
8. FPE	−.178	.163	−.084	.060	−.056	−.019	.265*	–	
9. Self-esteem	.137	−.021	.111	−.089	.120	.083	−.402**	−.383**	–

*Note.* *significant (*p* < .05) correlation between variables. **significant (*p* < .01) correlation between variables

### Event-related brain potentials

#### FRN

**Fig. 4:**
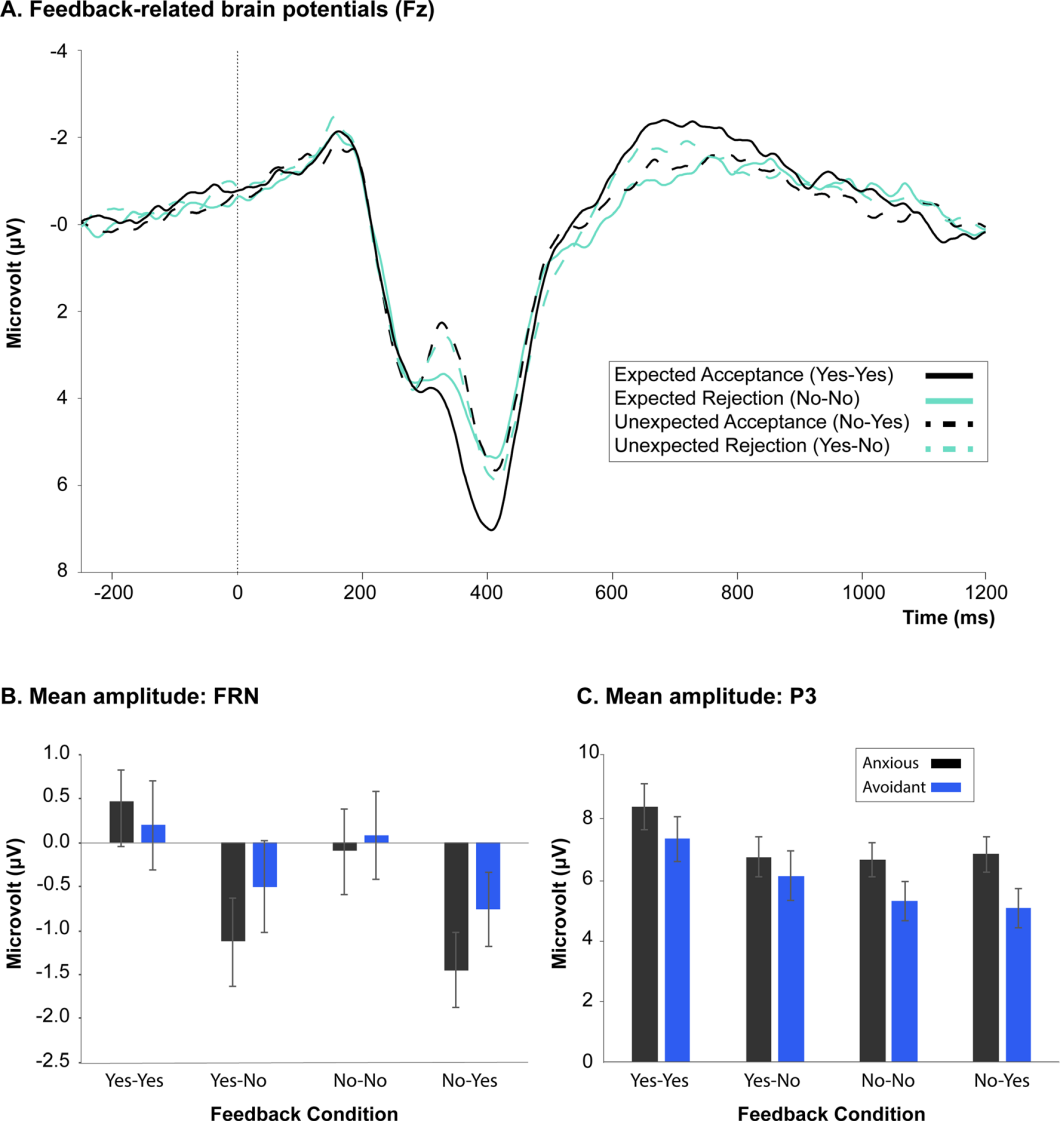
Feedback-related brain potentials elicited by social evaluative feedback. **a** Grand-averaged ERP for all participants per feedback condition. **b** Mean amplitude per subgroup for the feedback-related negativity (FRN). **c** Mean amplitude for the P3 component per subgroup. (Color figure online)

The mixed-design ANOVA yielded a main effect of Expectancy, *F*(1, 64) = 28.47, *p* < .001, η_p_^2^ = .311. FRN amplitude was significantly larger for unexpected than expected feedback (mean difference = −1.12 μV, *SEM* = .21). No significant main effect of Subgroup was observed *F*(1, 64) = .309, *p* = .580, η_p_^2^ = .005, nor did we find any significant other main/interaction effects. FRN results are depicted in Fig. 4.

#### P3

The mixed-design ANOVA on P3 amplitude showed a main effect of Expectancy, *F*(1, 64) = 8.75, *p* = .004, η_p_^2^ = .122, and Valence, *F*(1, 64) = 11.91, *p* = .001, η_p_^2^ = .159. These main effects were included into a significant Expectancy × Valence interaction, *F*(1, 64) = 15.96, *p* < .001, η_p_^2^ = .202. Post hoc comparisons revealed that P3 amplitude was significantly largest in the expected social acceptance condition (Yes-Yes) relative to all other conditions (Yes-No, *p* < .001; No-No, *p* < .001; No-Yes, *p* < .001). The main effect of Subgroup was not significant, *F*(1, 64) = 1.88, *p* = .175, η_p_^2^ = .029, nor did we find any significant interaction effects (*p*s > .05). The P3 results are depicted in Figure 4.^4^

## Discussion

The current study examined whether individual differences in personality constructs related to social evaluation (i.e., people with an anxious vs. avoidant profile on the constructs of attachment style, fear of negative/positive evaluation, and self-esteem) modulate theta power reactivity to unexpected social rejection feedback. The social judgment task was used to elicit feelings of social rejection and community structure analysis to subtract subgroups from our data based on the personality constructs. Two subgroups could be distinguished in our data set. One subgroup displayed an anxious profile, characterized by low scores on attachment-related avoidance and high scores on attachment-related anxiety and fear of negative evaluation. The other subgroup displayed an avoidant profile, characterized by high scores on attachment-related avoidance, and low scores on attachment-related anxiety and fear of negative evaluation. Unexpectedly, these subgroups did not differ in their FM-theta power reactivity to social evaluative feedback. In both groups, theta power was significantly highest in the unexpected rejection condition, which corroborates previous findings using this paradigm (van der Molen et al., 2017).

To our knowledge, the current study is the first to have used a network-theoretical approach to delineate personality profiles sensitive to social evaluation. This approach is particularly useful in fractionating the psychological symptoms or constructs implicated in various mental disorders with known sensitivity toward social disconnection. Our results thus add to the growing field in which network theoretical approaches are employed to understand how the clustering of symptoms might aid in a better understanding of psychological disorders (e.g., social anxiety disorder, depression; Fried et al., 2017; Heeren & McNally, 2016; Hoorelbeke, Marchetti, De Schryver, & Koster, 2016; Wichers, 2014). Our current results revealed that individual differences in attachment-related avoidance and anxiety were important in distinguishing between the two subgroup profiles. By including additional personality constructs relevant to social connectedness (e.g., fear of negative evaluation, self-esteem), community structure analysis furthermore suggested differences in personality traits of participants within the subgroups. Namely, participants who scored high on attachment-related anxiety also scored high on fear of negative evaluation, suggesting that these individuals might be more sensitive to signs of rejection or abandonment by others, and fear social rejection more. Whereas participants who scored high on attachment-related avoidance reported significantly lower levels of fear of negative evaluation, the fact that subgroup differences were observed for self-reported fear of negative evaluation and not positive evaluation underscores that the fear of scrutiny by others was a driving factor in distinguishing between subgroups.

The subgroups furthermore differed regarding their behavior on the SJP. The anxious subgroup was significantly slower in providing their predictions when they believed that peers disliked them. This finding is in accord with a previous study using this paradigm in which the level of FNE was positively correlated with response times during peer-rejection predictions (van der Molen et al., 2013). This was interpreted to suggest a greater degree of uncertainty, resulting in more time needed to predict the feedback outcome. In the current study, the subgroup difference in response time associated with peer-rejection predictions could be related to a similar process. That is, since participants in the anxious subgroup were generally optimistic about the social evaluative outcome (i.e., as reflected by the optimism bias in peer-feedback predictions), they might have been more uncertain when they decided peers might not have liked them. Further, their increased fear of negative evaluation might have increased their cognitive distress in the face of this uncertainty. Indeed, “intolerance of uncertainty” was found to be an important mediator between attachment-related anxiety and worrying (Wright, Clark, Rock, & Coventry, 2017) and has been shown to play an important role in anxiety and chronic worrying (Dugas, Buhr, & Ladouceur, 2004). Moreover, cognitive distress in response to uncertainty was previously associated with slower reaction time (Jackson, Nelson, & Hajcak, 2016). It should be noted that intolerance of uncertainty was not assessed in the current study via a direct measure and is not confined to anxiety and worrying (Shihata, McEvoy, Mullan, & Carleton, 2016; Wright et al., 2017). We do, however, suspect that it might have played a more significant role in the anxious subgroup due to their increased fear of being negatively evaluated by peers. Moreover, previous research suggests that people high in attachment-related avoidance often adopt “preemptive” strategies to avoid getting hurt in social interactions (Fraley & Brumbaugh, 2007). For example, avoidantly attached people often choose not to involve themselves in relationships with others. They view others more negatively, and adopt a negative attitude towards other people (Fraley & Brumbaugh, 2007). In general, one could argue that people with a more avoidant attitude towards social evaluation will therefore be less concerned about the outcome, which might result in less time providing their feedback predictions. In the current study, this might be reflected by the avoidant subgroup’s lowered expectancies to receive social acceptance feedback, and the absence of a discrepancy in response times associated with feedback predictions, suggesting an overall more disengaged attitude and decreased concern towards how others perceive them.

At the neural level, both subgroups reacted in a similar fashion to the presentation of social evaluative feedback. Corroborating previous findings (van der Molen et al., 2017), we observed that theta power was highest in response to unexpected social rejection feedback. Thus, although subjective ratings on the personality questionnaires might suggest a sensitivity toward signs of social rejection, the objective responses toward such stimuli in the brain are similar in individuals that fear rejection relative to those who do not. It was expected that individuals high in attachment-related anxiety and fear of negative evaluation would show increased responsivity of the neural alarm system that picks up cues communicating social threat (Clark & McManus, 2002; DeWall et al., 2012; Gillath et al., 2005; Guyer et al., 2008; van der Molen et al., 2013), whereas this system would show a blunted response in avoidantly attached individuals (Campbell et al., 2005; DeWall et al., 2012; Fraley & Brumbaugh, 2007; Mikulincer, 2016; Vrticka & Vuilleumier, 2012). However, no differences in theta oscillatory reactivity (as a putative index of the alarm system) were observed. This speaks to the notion that this theta response to unexpected social rejection feedback is a common and automatized process (van der Molen et al., 2017) and social rejection feedback might elicit a universal saliency response. In this sense, receiving unexpected rejection feedback might be equally important for both subgroups since it might yield a threat to social connectedness and requires appropriate actions to maintain social bonds (Baumeister & Leary, 1995). The fact that theta power was strongest in response to unexpected rejection feedback also meshes with the notion that FM-theta oscillations play a critical role in exerting cognitive control during situations that might be most uncertain (Cavanagh & Shackman, 2015), rendering the investment of this control as advantageous in the service of optimizing decision-making processes (Shenhav et al., 2013; Shenhav et al., 2016). That is, compared to the other feedback conditions, unexpected rejection feedback would be most compromising to the individual due to the high level of cognitive conflict and negative valence of this feedback. As such, increased engagement of FM-theta power might prepare the individual to undertake appropriate actions to maintain/gain social connectedness.

As for the feedback-related brain potentials examined in this study, we observed that the FRN was largest in response to unexpected feedback, regardless of valence. This is in accord with prior ERP studies using this paradigm (Dekkers et al., 2015; van der Molen et al., 2017; van der Molen et al., 2013), suggesting a differential sensitivity of phase-locked (ERPs) versus non-phase-locked (theta power) neural reactivity towards the processing of social evaluative feedback. In addition, the P3 was largest in response to expected social acceptance feedback, and this effect was most dominantly observed at the anterior midline. This finding is in line with prior results (van der Veen, van der Molen, van der Molen, & Franken, 2016; van der Veen et al., 2014) and has been taken to reflect the motivational relevance and potential reward conveyed by expected social acceptance feedback. That is, individuals might be particularly biased toward seeing their predictions to be “liked” by others confirmed (van der Veen et al., 2014). This dovetails with the idea that prefeedback (anticipatory) motivational states might contribute to generation of reward-related neural activity as indexed by the P300 (Pornpattananangkul & Nusslock, 2015; Threadgill & Gable, 2016). Indeed, anticipatory neural activity during social feedback expectations was found to be higher for anticipated social acceptance than rejection (van der Molen et al., 2013), although a causal relationship between prefeedback versus postfeedback neural processes remains to be established.

Notably, we did not observe any individual differences in neural response to social evaluative feedback. This might be due to the alleged universal saliency of these very early brain responses to feedback (300–500 ms after feedback onset). Possibly, individual differences in emotional responsivity to social feedback processing are tracked by slow-cortical potentials at later processing stages (van Noordt, White, Wu, Mayes, & Crowley, 2015). Although we did not find evidence of FM-theta power reactivity (nor other frequency modulations) during later parts of the feedback-processing window, future investigations could alter the design of the paradigm to examine repeated effects of acceptance versus rejection (e.g., in block-designs), as this approach is known to increase emotional distress in response to the threat of social disconnection (Cristofori et al., 2013; Crowley, Wu, Molfese, & Mayes, 2010; Sreekrishnan et al., 2014; van Noordt et al., 2015).

A few limitations to the current study could potentially have masked the hypothesized individual differences in theta reactivity to social evaluative feedback. First, our sample comprises undergraduate female students without (sub)clinical symptoms of psychopathology. That is, the subgroups’ ECR scores for the anxiety subscale were within range of previously published normative data of two large student-based samples with similar age ranges (Alonso-Arbiol, Balluerka, Shaver, & Gillath, 2008). Scores for the avoidance subscale were below this normative range. The subgroups’ average score was below the clinical threshold of the fear of negative evaluation questionnaire (>38; Carleton et al., 2011) and above the clinical threshold of the self-esteem questionnaire (<15; Roth, Decker, Herzberg, & Brahler, 2008). We did expect that low self-esteem would be related to increased attachment-related anxiety and fear of negative evaluation, and that at a neural level this would relate to increased responsivity of the neural alarm system (Somerville et al., 2010). Although the scores of our subgroups differed enough to account for behavioral group contrasts, the lack of extreme scores within our groups likely explains the similar neural alarm mechanism to social threat in both groups. A related issue is the relative low Q index observed in the current study that provides an indication of the robustness of the subgroup detection. Although a Q index larger than zero suggests a division between subgroups that exceeds chance level (Newman, 2004), similarity between groups was still present, particularly on the self-esteem and fear of positive evaluation constructs. Future studies should preferably include participants that display more extreme scores on the currently studied personality constructs, as this could contribute to the distinctiveness of subgroups (Fortunato & Barthelemy, 2007), as well as detecting individual differences in the neural response to social evaluative feedback. Further, in this study we focused on females due to their heightened sensitivity to social evaluation (Stroud et al., 2002). However, it would be interesting to examine (1) whether the clustering of personality constructs related to social evaluative processes is similar in males and females, and (2) if gender differences exist in the neural responsivity to social evaluative feedback. Such an approach would undoubtedly contribute to improved characterization of psychological disorders. Lastly, our outcomes have possibly been influenced by changes in menstrual cycle and the use of contraceptives, since both are known to affect brain activity and behavior during social and cognitive tasks (McEwen & Milner, 2017; Warren, Gurvich, Worsley, & Kulkarni, 2014). In the future, we aim to deal with this by systematically controlling for these hormonal influences.

In conclusion, using a data-driven approach to delineate distinct personality profiles relevant to social evaluative processes, we were able to show that attachment-related avoidance and attachment-related anxiety are related to fear of negative evaluation in a different manner. In particularly, females that seem anxious about the quality of close relationships display increased fear of negative evaluation. The opposite effect is found in avoidantly attached females. These findings underscore that using community structure analyses is an attractive method to test associations between psychological constructs in an intrinsic manner. By examining these personality constructs for the first time vis-à-vis the neural reactivity in response to social evaluative feedback, we demonstrate that unexpected rejection feedback elicits a significant increase in FM-theta power. A finding that is most likely indicative of a threat to social connectedness.

## Electronic supplementary material

ESM 1 (DOC 33 kb)

## Footnotes

1 A paired-samples *t* test was performed to compare the average score for both ECR scales for the first and second administration time point. No differences were observed between scores of these two time points for the Avoidance scale (*p* = .217) and the Anxiety scale (*p* = .780). Therefore, for both scales, the average score of both time points was entered into the community structure analysis.

2 Exploratively, the P3 was also examined for data of both Fz and Pz in a mixed-design Site × Valence × Congruency ANOVA, with subgroup as between-subjects factor. This analysis indicated that P3 amplitudes were largest at Pz; however, this analysis confirmed that the effects of feedback manipulations were similar, albeit less prominent, at the posterior midline.

3 Similar correlations were calculated between the feedback-related brain potentials (FRN and P3) and the self-report measures, but these correlations did not reach statistical significance.

4 P3 analyses yielded similar results at the Pz electrode, but only the contrast between expected acceptance feedback and unexpected acceptance feedback was significant (Bonferroni corrected).
