# Supplementary material for: Community structure analysis of rejection sensitive personality profiles: A common neural response to social evaluative threat?
Source: Cogn Affect Behav Neurosci. 2018 Apr 12;18(3):581–95. doi: 10.3758/s13415-018-0589-1 (PMC5962625; doi:10.3758/s13415-018-0589-1)
Supplement: Supplementary file 1 — (DOC 33 kb) [file 13415_2018_589_MOESM1_ESM.doc]

**Supplementary data**

**Behavioral results**

Participants were slightly biased in their expectancies of the outcome of social evaluation. That is, participants predicted social acceptance feedback on 54% (*SD* = 8.78) of the trials, which differed significantly from 50%, *t*(64) = 3.83, *p* < .001. This is consistent with previous studies, showing similar positivity bias outcomes (Dekkers et al., 2015; van der Veen et al., 2016; van der Veen et al., 2014). A paired samples t-test indicated that the speed of judgments did not significantly differ between social acceptance and rejection expectancies (mean difference = 0.32 ms, *t*(64) = 1.87, *p* = 0.066). The average number of predicted social acceptance feedback (“Yes”) trials was 80.71 (*SD* = 13.07) and average response time for these predictions was 1407.69 ms (*SD* = 283.68 ms). The average number of predicted social rejection feedback (“No”) trials was 68.31 (*SD* = 13.15) and the average response time for these predictions was 1440.01 ms (*SD* = 307.27 ms). A paired samples t-test indicated that the assessed amount of acceptance feedback was significantly higher before the start of the SJP than after the SJP (mean difference = 20.42, *t*(63) = 13.28, *p* < .001). Average scores of self-report measures for the total sample are presented in Table 1.

**P3 results**

A repeated measures ANOVA on the P3 results with Site (2 levels: Fz, Pz), Valence (two levels: Acceptance, Rejection), and Congruency (2 levels: expected, unexpected) as within-subject factors, and Subgroup (2 levels: Anxious, Avoidant) as between-subjects factor. A main effect of Site was observed, indicating that P3 amplitudes were largest at the Pz electrode, *F*(1, 64) = 23.11, *p* < .0001, *η^2^_p_* = .27. A significant three-way interaction between Site x Valence x Congruency was observed, *F*(1, 64) = 5.18, *p* = .026, *η^2^_p_* = .08. Follow-up tests revealed that for Fz, P3 amplitude was significantly largest for the expected acceptance condition vs. the other feedback conditions (all *p*s < .0001). At the P3 site, P3 responses to expected acceptance feedback were only significantly larger in response to unexpected acceptance feedback (*p* = .001, Bonferroni corrected).
